# First results from a high-resolution small animal PET insert for PET/MRI imaging

**DOI:** 10.1186/2197-7364-2-S1-A54

**Published:** 2015-05-18

**Authors:** Andrew Goertzen, Greg Stortz, Jonathan Thiessen, Daryl Bishop, Muhammad Salman Khan, Piotr Kozlowski, Fabrice Retiere, Graham Schellenberg, Vesna Sossi, Christopher Thompson

**Affiliations:** University of Manitoba, Canada; University of British Columbia, Canada; Lawson Health Research Institute, Canada; TRIUMF, Canada; Montreal Neurological Institute, Canada

We have recently completed construction of a high resolution small animal PET insert designed for operation inside a Bruker 7T MRI. The PET insert is designed to achieve a 1 mm spatial resolution in the centre of its field of view (FOV) and fit within the 114 mm inner diameter of the Bruker BGA-12S gradient coil while accommodating the Bruker 35 mm volume RF coil (outer diameter 60 mm). The PET insert is a ring geometry with a single ring of 16 detectors. Each detector uses a dual layer offset (DLO) LYSO scintillator array (bottom/top layer: 22x10/21x9 of 1.2x1.2x6/4 mm crystals, 409 crystals per block), with total axial extent of 28.3 mm, readout by two SensL SPMArray4B SiPM arrays. The detector outputs are multiplexed to four signals using a custom readout board and digitized using the OpenPET data acquisition platform. Detector flood image quality is sufficient to resolve >99% of the crystals in the system. The average energy resolution of the 6544 crystals is 11.94%+/-1.77% at 511keV. MR compatibility testing of the complete PET system conducted with a 7T Bruker Avance III MRI showed that the operating PET insert had no effect on MRI image homogeneity and only a small effect on EPI signal to noise ratio (SNR) (-15%). Initial PET data were collected using a Ge68 line source with the PET system on the benchtop. For this first acquisition, the OpenPET system was operating in oscilloscope mode, limiting the total singles event rate to 18kcps. The sinogram and initial reconstructed images showed no obvious artefacts. We have recently implemented an OpenPET firmware upgrade that will support a singles rate of 280kcps; this will allow us to acquire first simultaneous phantom and mouse PET/MR images.

